# Influence of Prior Imaging Information on Diagnostic Accuracy for Focal Skeletal Processes—A Retrospective Analysis of the Consistency between Biopsy-Verified Imaging Diagnoses

**DOI:** 10.3390/diagnostics12071735

**Published:** 2022-07-17

**Authors:** Mine Benedicte Lange, Lars J. Petersen, Mads Lausen, Niels Henrik Bruun, Michael Bachmann Nielsen, Helle D. Zacho

**Affiliations:** 1 Department of Radiology, North Zealand Hospital, 3400 Hilleroed, Denmark; 2 Department of Diagnostic Imaging, North Zealand Hospital, 3400 Hilleroed, Denmark; 3 Department of Clinical Medicine, Aalborg University, 9000 Aalborg, Denmark; ljp@dadlnet.dk (L.J.P.); h.zacho@rn.dk (H.D.Z.); 4 Department of Nuclear Medicine, Clinical Cancer Research Center, Aalborg University Hospital, 9000 Aalborg, Denmark; 5 Department of Clinical Microbiology, Copenhagen University Hospital, Rigshospitalet, 2100 Copenhagen, Denmark; madsln@biosustain.dtu.dk; 6 Department of Research and Statistics, Aalborg University Hospital, 9000 Aalborg, Denmark; nbru@rn.dk; 7 Department of Radiology, Copenhagen University Hospital, Rigshospitalet, 2100 Copenhagen, Denmark; michael.bachmann.nielsen@regionh.dk

**Keywords:** bone, cancer, metastasis, tumor, biopsy, diagnostic accuracy, medical imaging, reports, prior imaging

## Abstract

Introduction: Comparing imaging examinations with those previously obtained is considered mandatory in imaging guidelines. To our knowledge, no studies are available on neither the influence, nor the sequence, of prior imaging and reports on diagnostic accuracy using biopsy as the reference standard. Such data are important to minimize diagnostic errors and to improve the preparation of diagnostic imaging guidelines. The aim of our study was to provide such data. Materials and methods: A retrospective cohort of 216 consecutive skeletal biopsies from patients with at least 2 different imaging modalities (X-ray, CT and MRI) performed within 6 months of biopsy was identified. The diagnostic accuracy of the individual imaging modality was assessed. Finally, the possible influence of the sequence of imaging modalities was investigated. Results: No significant difference in the accuracy of the imaging modalities was shown, being preceded by another imaging modality or not. However, the sequence analyses indicate sequential biases, particularly if MRI was the first imaging modality. Conclusion: The sequence of the imaging modalities seems to influence the diagnostic accuracy against a pathology reference standard. Further studies are needed to establish evidence-based guidelines for the strategy of using previous imaging and reports to improve diagnostic accuracy.

## 1. Introduction

Radiology is one of the specialties most liable to claims of diagnostic negligence, which can be defined as errors resulting in incorrect, delayed, or missed diagnoses [1,2,3]. Several studies have investigated the incidence and causes of medical errors, but such analyses remain challenging due to the lack of effective methods for measurement and limited sources of reliable data [4].

A diagnostic report consists of the complete detection and accurate diagnosis of all abnormalities in an imaging examination and at the same time as accurately as possible to distinguish which lesions can be safely ignored from those requiring additional workup or biopsy, most often described as either benign or possible malignant. The average error rate among radiologists has been shown to be approximately 30%, referring to images as part of a set of unknowns with proven pathology, a prevalence that has remained unchanged since it was first estimated in the 1960s [5,6,7]. The etiology of radiological error is multifactorial, including failure to compare with prior imaging and reports, bias, poor technique, failures of perception, lack of knowledge, fatigue, noise, and misjudgments [8]. More than 70% of errors are perceptual, whereas fewer than 30% are cognitive [5]. One study showed that radiologists disagreed with each other more than 30% of the time and with themselves more than 25% of the time [9]. It is considered without debate to be the standard of care by the radiology and the non-radiology medical communities that radiologists must compare new imaging examinations with those obtained previously [10,11,12,13,14,15,16]. Failure to consult prior radiologic studies has been shown to represent 5% of the explanation for missed findings [5,7,10,17]. Previous images are subjectively judged to be more valuable than imaging reports for documenting disease progression on conventional X-ray images [18,19]. Studies have shown that if one looks at a prior negative report before looking at imaging studies, there is a greater chance of missing a significant abnormality than by looking at the imaging studies first [5]. It has also been shown that radiological diagnoses made with adequate clinical information are more accurate than those made without clinical information [20,21,22,23]. However, to the best of our knowledge, no studies have investigated the influence, or the sequence, of prior imaging and reports on diagnostic accuracy using biopsy as the reference standard. Such data are of great importance not only to minimize diagnostic errors but also to improve the preparation of diagnostic imaging guidelines based upon diagnostic accuracy and cost-effectiveness.

The purpose of our study was to investigate whether the diagnostic accuracy of the detection of skeletal malignancies, proven malign or benign by subsequent biopsy, is affected by prior imaging examinations and their mutual sequences.

## 2. Materials and Methods

### 2.1. Collection of Skeletal Biopsies

The study was conducted as a retrospective consecutive cohort study. Bone biopsies were identified by performing a computer search of pathology samples representing bone material registered by SNOMED (Systematized Nomenclature of Medicine) T10* and T11* codes for skeletal cytology and histology biopsies from 1 January 2011 to 31 July 2013, at the Department of Pathology, and each biopsy was identified by a unique social security number [24]. The eligibility criteria for a biopsy to be included in the analysis were conclusive pathology results performed by a board-certified pathologist. The biopsies were processed and analyzed in accordance with institutional practice, and immunohistochemical examination was applied when relevant. If several biopsies were obtained from the same anatomical region within a period of 6 months and one of these biopsies showed malignancy, the lesion was classified as malignant. If repeated biopsies showed a benign condition, the first biopsy was used.

Each pathology report was reviewed by two readers and classified as benign, malignant, or inconclusive. In the case of inconsistency, a board-certified pathologist assisted with a conclusion.

**Figure 1:**
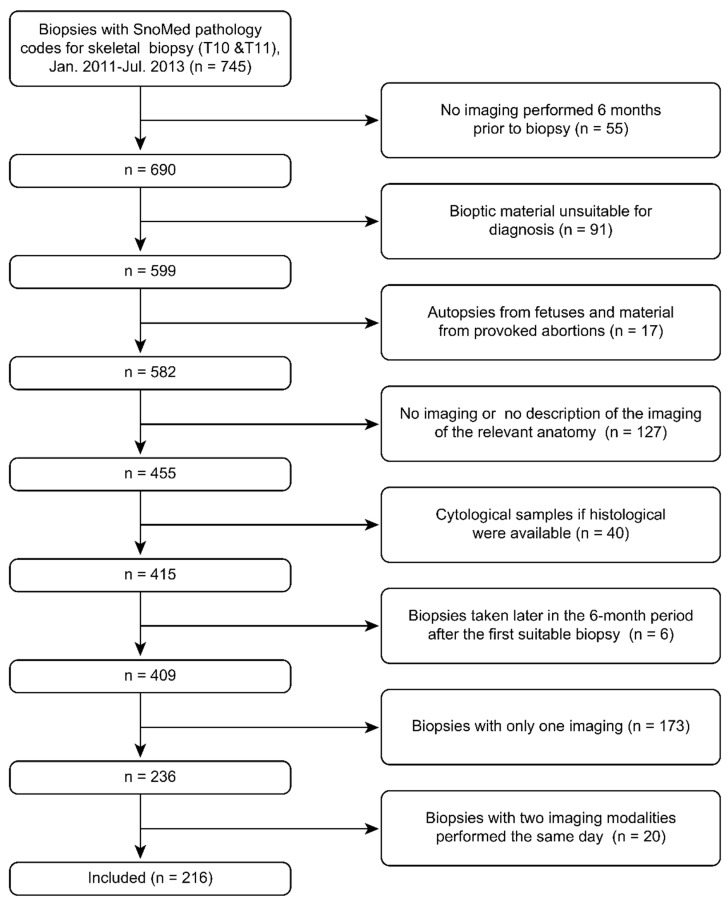
Flow chart of study material.

The baseline dataset was used for two previously published articles, and the exclusion criterion for the present study was biopsies performed with less than two different imaging modalities six months prior to the biopsy (Figure 1) [24,25].

### 2.2. Imaging

Diagnostic imaging included X-ray, computed tomography (CT) and magnetic resonance imaging (MRI). X-ray imaging was performed by digital radiography, and the CT scans were performed on either a GE (GE Lightspeed VCT, 64 slice, GE LightSpeed Pro, 32 slice, GE Discovery 750HD, General Electrics, Milwaukee, WI, USA) or a Siemens (SIEMENS Definition Flash Siemens AG, 128 slice) scanner. MRI scans were performed on a 1.5 T MR scanner (Discovery MR450, General Electrics, Milwaukee, WI, USA). The MRI image sequences were T1, T2 and STIR, of which at least one sequence was axial on the bone involved; contrast was only given in cases of soft tissue involvement, which was decided in each case by a radiology specialist. Bone scintigraphy (BS), single photon emission computed tomography CT (SPECT/CT), 18F-fluorodeoxyglucose positron emission tomography (FDG-PET/CT) and ultrasound (US) were excluded due the low number of combinations of those with another.

*All radiology imaging procedures were performed in accordance with institutional guidelines (no experimental imaging investigations were included in the analysis), and the written reports were reviewed by two independent reviewers who, based upon the description and conclusions in the original text, classified the described lesion as malignant, benign, or inconclusive. In cases of disagreement after individual reading, the readers reached consensus for each imaging report without the need for a third-party arbitrator. The radiologists had access to an Electronical Patient Journal charts (EPJ—Clinical Suite, CSC Scandihealth A/S) for any relevant journal notes in case they needed more information than was stated in the referral.*

### 2.3. Statistics

Statistical analysis was performed by using Stata 17 (StataCorp LLC 2021) and the Stata package matrix tools [26]. Sensitivity, specificity, prevalence, accuracy, positive predictive value (PPV) and negative predictive value (NPV) with 95% confidence intervals were calculated for each imaging modality without taking the imaging sequence into consideration. Then, it was calculated for pairs of imaging modalities, such as X-ray/CT and CT/X-ray, and by doing so, not all X-ray stand-alone values were included to minimize the bias that only one imaging was performed as opposed to two. The diagnostic properties of one modality (CT, MRI, and X-ray) when used as the first imaging modality were compared with the diagnostic properties of the modality when it was preceded by another modality using Fisher’s exact test. It should be noted that the numbers in some of the subgroups may be too low to detect significant differences. Finally, the effect of the imaging sequence was examined among patients with a malignant biopsy diagnosis and with a benign biopsy diagnosis; due to the small number in each group, only descriptive statistics were used.

### 2.4. Approval

This retrospective study did not require ethical approval or informed consent in accordance with national legislation. The Danish Data Protection Agency approved the study and provided permission to access medical files for the purpose of the study.

## 3. Results

### 3.1. Baseline Data

Most of the biopsies were malignant (Table 1), with lung cancer (31%), breast cancer (19%), multiple myeloma (12%) and lymphoma (11%) being the most frequent types of cancer. The benign lesions were mainly characterized as inflammation, fibrosis, osteochondroma, degenerative changes, nonspecific reactive changes, necrosis, and fracture. There was a slight predominance of males over females, and the spine was the most common anatomical localization of bone biopsy. The three included imaging modalities were almost equally represented (Table 1). Most biopsies (67%) had two imaging modalities performed 6 months prior to biopsy, 30% had three imaging modalities performed and 3% had four imaging modalities performed (details are provided in Table S1).

MRI was shown to have the highest accuracy, followed by CT and X-ray when the sequence of imaging was not taken into consideration (Table 2). MRI also showed the highest sensitivity and NPV, whereas X-ray proved to have the highest specificity and CT the highest PPV (Table 2).

### 3.2. Sequence Analysis

Taking the sequence of imaging modalities into account, no significant difference in accuracy within each imaging modality was seen when preceded by another imaging modality or not (Table 3, Table 4 and Table 5), except for a decrease in CT specificity and PPV when preceded by MRI (Table 3. Despite the lack of difference in overall accuracy, an interesting pattern of observations was seen when examining the sequences for imaging divided by malignant and benign biopsies.

**Figure 2:**
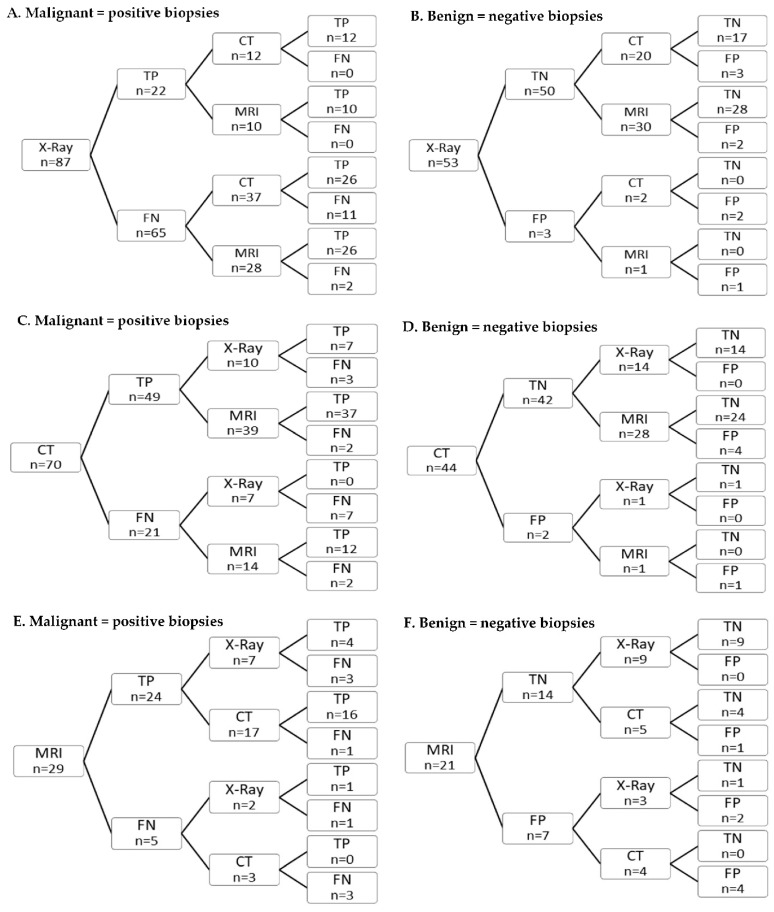
Sequence analyses. (**A**) Diagnostic results for malignant (positive) biopsies when X-ray is performed as the first modality (TP = true positive, FN = false negative). (**B**) Diagnostic results for benign (negative) biopsies when X-ray is performed as the first modality (TN = true negative; FP = false positive). (**C**) Diagnostic results for malignant (positive) biopsies when CT is performed as the first modality (TP = true positive, FN = false negative). (**D**) Diagnostic results for benign (negative) biopsies when CT is performed as the first modality (TN = true negative; FP = false positive). (**E**) Diagnostic results for malignant (positive) biopsies when MRI is performed as the first modality (TP = true positive, FN = false negative). (**F**) Diagnostic results for benign (negative) biopsies when MRI is performed as the first modality (TN = true negative; FP = false positive).

Among malignant (positive) biopsies, it was seen that if X-ray was false negative (75%) and used as the first imaging modality, only 7% of the subsequent MRI and 30% of the subsequent CT imaging were false negative (Figure 2A), whereas if MRI was false negative (17%) and conducted as the first imaging modality, 100% of the following CT scans were false negative as well (Figure 2E). Likewise, among biopsies with a benign (negative) histology, if MRI was false-positive (33%), 100% of the subsequent CT imaging was also false-positive (Figure 2F). Figure 2C demonstrates that when CT scans were false negative (30%), 100% of the subsequent X-ray examinations were false negative, whereas this was only the case for 14% of the subsequent MRI. For the few false-positive X-ray and CT ex.

## 4. Discussion

Without taking the imaging sequence into consideration, MRI was shown to have the highest accuracy, followed by CT and X-ray, and MRI also showed the highest sensitivity and NPV, whereas X-ray proved to have the highest specificity and CT had the highest PPV (Table 2). These findings are consistent with previously published data, out of which one study is against a pathology proven reference [24,27,28,29,30]. These imaging characteristics are generally well recognized by radiologists.

Taking image sequence into consideration, our results show that there is no significant difference to prove that the diagnostic accuracy of X-ray, CT or MRI is influenced by access to prior imaging examinations and reports of one of the other modalities. This finding is controversial because it is not in accordance with previous studies and present guidelines, describing the importance of always comparing actual imaging with previous examinations and reports [5,7,10,11,12,13,14,15,17,18,19]. There might be several explanations for our findings.

Primarily, the lack of significance may be caused by the small subgroups. Second, the lack of difference in accuracy could cover the two opposing situations, as when MRI is the first imaging modality, it can either be correct or incorrect. According to our sequence analysis, when the MRI is correct, then the subsequent CT or X-ray is more likely to be correct, and when MRI is incorrect, then the subsequent CT or X-ray is incorrect in more than 80% of the situations. These two situations might balance each other so that the accuracy does not change significantly compared to whether a modality is preceded by MRI.

When X-ray is the first modality, 75% are expectedly false negatives, but only 7% of the subsequent MRI and 30% of the subsequent CT examinations are false negatives as well, which could indicate that X-ray results are rightfully not considered to have a high sensitivity and therefore do not influence the reader’s evaluation of the second imaging much. When CT is the first modality, 30% are false negatives, and then all the following 7 X-ray examinations are negative, whereas only 14% of the subsequent MRIs are negative as well. CT has a higher accuracy than X-ray, and therefore, the reader might tend to attach greater value to the results from CT than those from the X-ray itself, whereas this is not the case for MRI compared to CT. When MRI is the first modality, only 17% are false negatives, with all subsequent CT scans being false negatives as well. Again, the reader might put more value on the previous MRI.

The specificities of X-ray (98.0) and CT (93.6) as stand-alone are high and decrease when preceded by MRI. On the contrary, the specificity increases for MRI (from 71.9 to 90.3) when preceded by an X-ray. Since X-ray specificity is known to be high, it might influence the reader of the consequent MRI scan.

One might conclude that the higher the diagnostic accuracy a given modality is known to have, the higher the bias of the diagnostic accuracy of the subsequent modalities will be and therefore that the sequence of the imaging modalities is important, especially if the diagnosis of the first modality is proven false. It has been shown previously that if one looks at a prior negative report before looking at imaging studies, there is a greater chance of missing a significant abnormality than by looking at the imaging studies first, but in these studies all imaging involved was X-ray and no other modality was included [5,17].

A direct comparison of the different imaging sequences to evaluate which sequence would be interesting for diagnosis and follow-up should be made with caution. It was not the purpose of our study; some groups are small, and we have not been able to prove any significant differences. MRI preceded by X-ray showed a sensitivity of 94.7 and a PPV of 92.3, slightly higher than MRI preceded by CT, showing a sensitivity of 92.5 and a PPV of 90.7. Since CT gives a higher radiation dosage and is more expensive than X-ray, you could speculate if X-ray followed by MRI would be the best strategy. This could make sense if you consider the bone lesion to be an isolated lesion, but since the malignant lesions represent metastases, you will most often need a CT scan to identify a primary tumor and/or to see if the skeletal lesion is the only metastasis present. The benefit of CT is that it is a whole-body examination, which is more readily available and inexpensive than whole-body MRI or whole-body fusion imaging techniques such as PET-CT or PET-MRI. Therefore, it would be impossible to avoid performing a CT scan in most cases. Further prospective research is necessary to clarify this topic.

To the best of our knowledge, no direct comparison of pathology-proven diagnostic accuracy, including X-ray, CT, or MRI, with or without previous imaging examinations and the sequence of those has been conducted. Such knowledge should be considered quite important, not only in everyday imaging reporting but also in cases of claims of medical negligence. We identified four studies investigating whether access to prior examinations was valuable. All studies compared plain radiographs with prior plain radiographs and were based on questionnaires completed by the interpreting radiologists on whether they found access to prior examinations to be valuable or not [12,13]. Nevertheless, all present guidelines emphasize the importance of comparison with prior diagnostic examinations and reports of any modality available; however, these recommendations do not seem to be evidence-based.

Our findings could indicate that guidelines for good practice of radiological imaging reading and reporting should point out the importance of the readers not being influenced too much by previous imaging, especially not if these are modalities that are usually considered to be more accurate than the current one and that biopsy should be considered the gold standard for a valid diagnosis [25]. In clinical practice, one should consider evaluating the present study without a prior review of previously available imaging studies. When an independent evaluation has been formed, you can look at the available previous studies. If these conflict with your assessment, you should consider whether you want to be influenced and if so, you could note this in the description.

In addition to the small number in some of our subgroups, there are other limitations to our study. Table S1 in the Supplement shows that 33% of the biopsies had 3 or 4 imaging scans performed, which is a bias to the results since modalities other than the one analyzed could influence the diagnostic accuracy. However, there was no significant difference between the diagnostic accuracy regardless of whether the imaging investigated was preceded by other modalities. Furthermore, the readers had access to clinical information via Clinical Suite, and we do not know how many actually received this clinical information, which is known to influence the diagnostic reports [20,21,22,23]. Finally, it has been shown that the localization of the lesion has an influence on the diagnostic accuracy, with MRI showing superior diagnostic properties in spine lesions, whereas in non-spine lesions, the accuracy of the imaging modalities is largely comparable [24]. In our study, the spine accounted for 55% of the localizations, extremities for 18 % and pelvis for 17 %. The limited sample size does not allow for subgroup analysis on localization, which might represent a limitation. In conclusion, our study demonstrates the contribution to the discussion of the possible influence of previous imaging and reporting on diagnostic accuracy and how this possible influence should be addressed in future guidelines for the interpretation and reporting of diagnostic imaging. New prospective studies on this topic are needed for this purpose.

## Figures and Tables

**Table 1:** Baseline demographics.

Variable	Value
Patients (n = 207)	
Male, n (%)	116 (56%)
Female, n (%)	91 (44%)
Age, median (range)	67 (1–93)
Biopsies (n = 216)	
Malignant, n (%)	132 (61%)
Benign, n (%)	84 (39%)
Biopsy specimen (n = 216)	
Cytological, n (%)	16 (8%)
Histological, n (%)	195 (90%)
Dissection, n (%)	5 (2%)
Imaging modalities performed (n = 464)	
X-ray, n (% of biopsies)	143 (66%)
CT, n (% of biopsies)	169 (78%)
MRI, n (% of biopsies)	152 (70%)
Localization of bone lesion (n = 216)	
Spine, n (%)	119 (55%)
Extremities, n (%)	39 (18%)
Pelvis, n (%)	36 (17%)
Thorax and shoulders, n (%)	19 (9%)
Head, n (%)	3 (1%)

**Table 2:** Sensitivity, specificity, accuracy, positive and negative predictive values (PPV, NPV) estimates of imaging techniques for detection of focal skeletal lesions.

	X-ray (n = 143)	CT (n = 169)	MRI (n = 152)
Sensitivity	31.3 (21.4–42.6)	73.5 (64.3–81.3)	92.1 (84.5–96.8)
Specificity	95.2 (86.7–99.0)	85.7 (73.8–93.6)	81.0 (69.1–89.8)
Accuracy	59.4 (50.9–67.6)	77.5 (70.5–83.6)	87.5 (81.2–92.3)
PPV	89.3 (71.8–97.7)	91.2 (83.4–96.1)	87.2 (78.8–93.2)
NPV	52.2 (42.7–61.6)	61.5 (49.8–72.3)	87.9 (76.7–95.01)

Note—95% exact confidence intervals for each imaging modality without taking the imaging sequence into consideration.

**Table 3:** Sensitivity, specificity, accuracy, PPV and NPV estimates (reported with 95% confidence intervals) for X-ray and CT without or with access to a preceding MRI.

	X-ray			CT		
	Not Preceded by MRI (n = 122)	Preceded by MRI (n = 21)	*p* Value	Not Preceded by MRI (n = 140)	Preceded by MRI (n = 29)	*p* Value
Sensitivity	28.2 (18.1–40.1)	55.6 (21.2–86.3)	0.13	72.0 (61.8–80.9)	80.0 (56.3–94.3)	0.58
Specificity	98.0 (89.6–100.0)	83.3 (51.6–97.9)	0.09	93.6 (82.5–98.7)	44.4 (13.7–78.8)	0.00
Accuracy	70.0 (63.1–76.3)	65.2 (42.7–83.6)	0.34	79.3 (71.6–85.7)	69.0 (49.2–84.7)	0.23
PPV	95.2 (76.2–99.9)	71.4 (29.0–96.3)	0.15	95.7 (88.0–99.1)	76.2 (52.8–91.8)	0.01
NPV	49.5 (39.4–59.6)	71.4 (41.9–91.6)	0.16	62.9 (50.5–74.1)	50.0 (15.7–84.3)	0.48

**Table 4:** Sensitivity, specificity, accuracy, PPV and NPV estimates (reported with 95% confidence intervals) for X-ray and MRI without or with access to a preceding CT.

	X-ray			MRI		
	Not Preceded by CT (n = 111)	Preceded by CT (n = 32)	*p* Value	Not Preceded by CT (n = 70)	Preceded by CT (n = 82)	*p* Value
Sensitivity	28.6 (17.9–41.3)	41.2 (18.4–67.1)	0.38	91.7 (77.5–98.2)	92.5 (81.8–97.9)	1.00
Specificity	93.8 (82.8–98.7)	100.0 (78.2–100.0)	1.00	79.4 (62.1–91.3)	82.8 (64.2–94.2)	1.00
Accuracy	56.8 (47.0–66.1)	68.8 (50.0–83.9)	0.84	85.7 (75.3–92.9)	89.0 (80.2–94.9)	0.63
PPV	85.7 (63.7–97.0)	100.0 (59.0–100.0)	0.55	82.5 (67.2–92.7)	90.7 (79.7–96.9)	0.35
NPV	50.0 (39.9–60.7)	60.0 (38.7–78.9)	0.50	90.0 (73.5–97.9)	85.7 (67.3–96.0)	0.70

**Table 5:** Sensitivity, specificity, accuracy, PPV and NPV estimates (reported with 95% confidence intervals) for CT and MRI without or with access to a preceding X-ray.

	CT			MRI		
	Not Preceded by X-ray (n = 98)	Preceded by X-ray (n = 71)	*p* Value	Not Preceded by X-ray (n = 83)	Preceded by X-ray (n = 69)	*p* Value
Sensitivity	70.3 (57.6–88.1)	77.6 (63.4–88.2)	0.52	90.2 (78.6–96.7)	94.7 (82.3–99.4)	0.69
Specificity	91.2 (76.3–98.1)	77.3 (54.6–92.2)	0.24	71.9 (53.3–86.3)	90.3 (74.2–98.0)	0.11
Accuracy	77.6 (68.0–65.4)	77.5 (66.0–86.5)	1.00	83.1 (73.3–90.5)	92.8 (83.9–97.6)	0.09
PPV	93.8 (82.8–98.7)	88.4 (74.9–96.1)	0.47	83.6 (71.2–92.2)	92.3 (79.1–98.4)	0.35
NPV	62.0 (47.2–75.3)	60.7 (40.6–78.5)	1.00	82.1 (63.1–93.9)	93.3 (77.9–99.2)	0.25

## Data Availability

Data supporting reported results can be found in a special locked folder with an excel sheet within our institution and can be provided if necessary.

## Supplementary Materials

The following supporting information can be downloaded at: https://www.mdpi.com/article/10.3390/diagnostics12071735/s1, Table S1: Modality sequence.
