# Supplementary material for: Influence of Prior Imaging Information on Diagnostic Accuracy for Focal Skeletal Processes—A Retrospective Analysis of the Consistency between Biopsy-Verified Imaging Diagnoses
Source: Diagnostics (Basel). 2022 Jul 17;12(7):1735. doi: 10.3390/diagnostics12071735 (PMC9319824; doi:10.3390/diagnostics12071735)
Supplement: Supplementary file 1 [file diagnostics-12-01735-s001.zip › diagnostics-1740349-supplementary.pdf]

Table S1 Supplements. Modality sequence

Modality sequence	Number of modalities per biopsy			Total
	**2**	**3**	**4**	
X-Ray/CT	25			**25**
X-Ray/CT/MRI		23		**23**
X-Ray/CT/MRI/PET-CT			1	**1**
X-Ray/CT/PET-CT		3		**3**
X-Ray/CT/PET-CT/MRI			2	**2**
X-Ray/MRI	24			**24**
X-Ray/MRI/CT		13		**13**
X-Ray/MRI/PET-CT		1		**1**
X-Ray/PET-CT	3			**3**
X-Ray/PET-CT/MRI		1		**1**
CT/MRI	39			**39**
CT/MRI/PET-CT		3		**3**
CT/MRI/X-Ray		7		**7**
CT/PET-CT	8			**8**
CT/PET-CT/MRI		2		**2**
CT/PET-CT/X-Ray		2		**2**
CT/PET-CT/X-Ray/MRI			1	**1**
CT/X-Ray	16			**16**
CT/X-Ray/MRI		3		**3**
MRI/CT	10			**10**
MRI/CT/PET-CT/X-Ray			1	**1**
MRI/CT/X-Ray		1		**1**
MRI/CT/ X-Ray/PET-CT			1	**1**
MRI/PET-CT	3			**3**
MRI/PET-CT/X-Ray		1		**1**
MRI/PET-CT/X-Ray/CT			1	**1**
MRI/X-Ray	6			**6**
MRI/X-Ray/CT		2		**2**
PET-CT/CT	3			**3**
PET-CT/CT/MRI		1		**1**
PET-CT/MRI	4			**4**
PET-CT/MRI/X-Ray		1		**1**
PET-CT/X-Ray	3			**3**
PET-CT/X-Ray/CT		1		**1**
**Total**	**144 (67%)**	**65 (30%)**	**7 (3 %)**	**216**
